# Supplementary material for: Insights into the Genomic Potential of a Methylocystis sp. from Amazonian Floodplain Sediments
Source: Microorganisms. 2022 Aug 30;10(9):1747. doi: 10.3390/microorganisms10091747 (PMC9506196; doi:10.3390/microorganisms10091747)
Supplement: Supplementary file 1 [file microorganisms-10-01747-s001.zip › microorganisms-1772199-supplementary.pdf]

Supplementary Material

Insights into the genomic potential of a *Methylocystis* sp. from Amazonian floodplain sediments

Júlia B. Gontijo*, Fabiana S. Paula, Andressa M. Venturini, Jéssica A. Mandro, Paul L.E. Bodelier and Siu M. Tsai

*Correspondence: juliabrandao@usp.br

1 Supplementary Figures

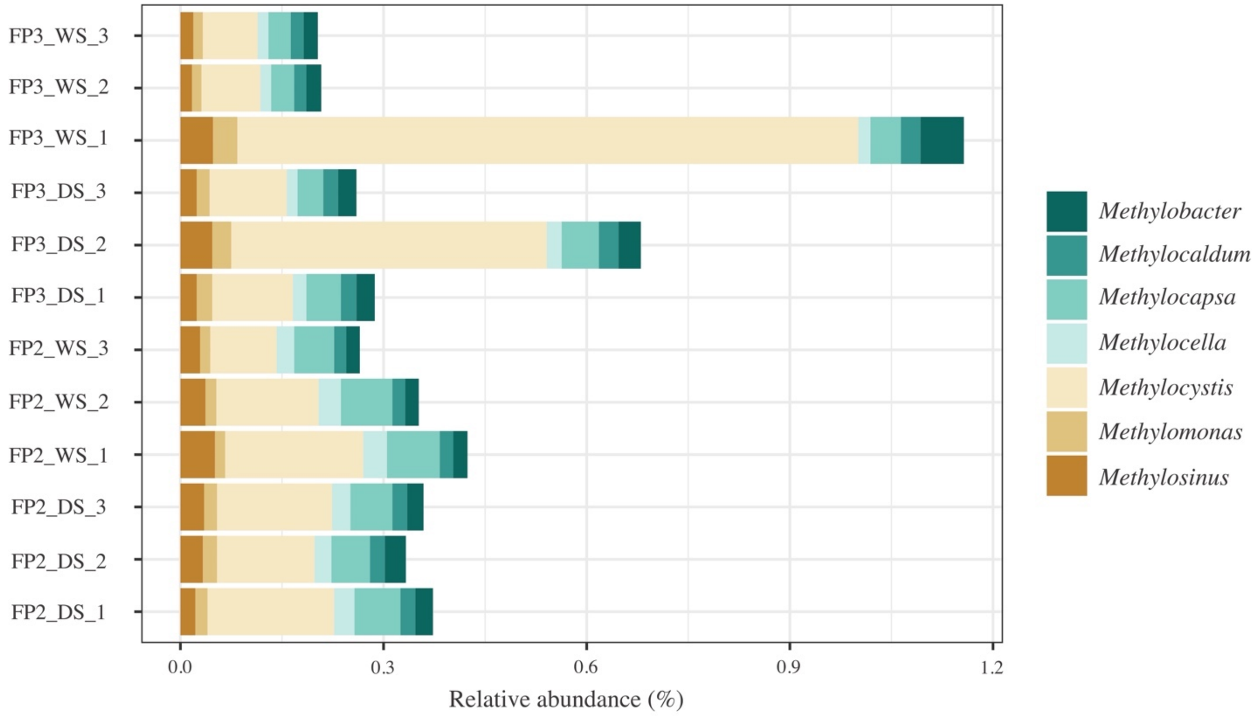

Supplementary Figure S1. Relative abundance of methanotrophs (> 0.01%) in the reads from the 12 metagenomic samples used for the bin.170_fp reconstruction. FP2 represents the floodplain located on the Amazonas River (2°28'11.2"S 54°38'49.9"W) and FP3 represents the floodplain located at the intersection between Amazonas and Tapajós Rivers (2°22'44.8"S 54°44'21.1"W). WS and DS represents the wet and dry seasons. The methanotrophic taxa were manually filtered considering the Methanotroph Commons database (http://www.methanotroph.org/wiki/taxonomy/).

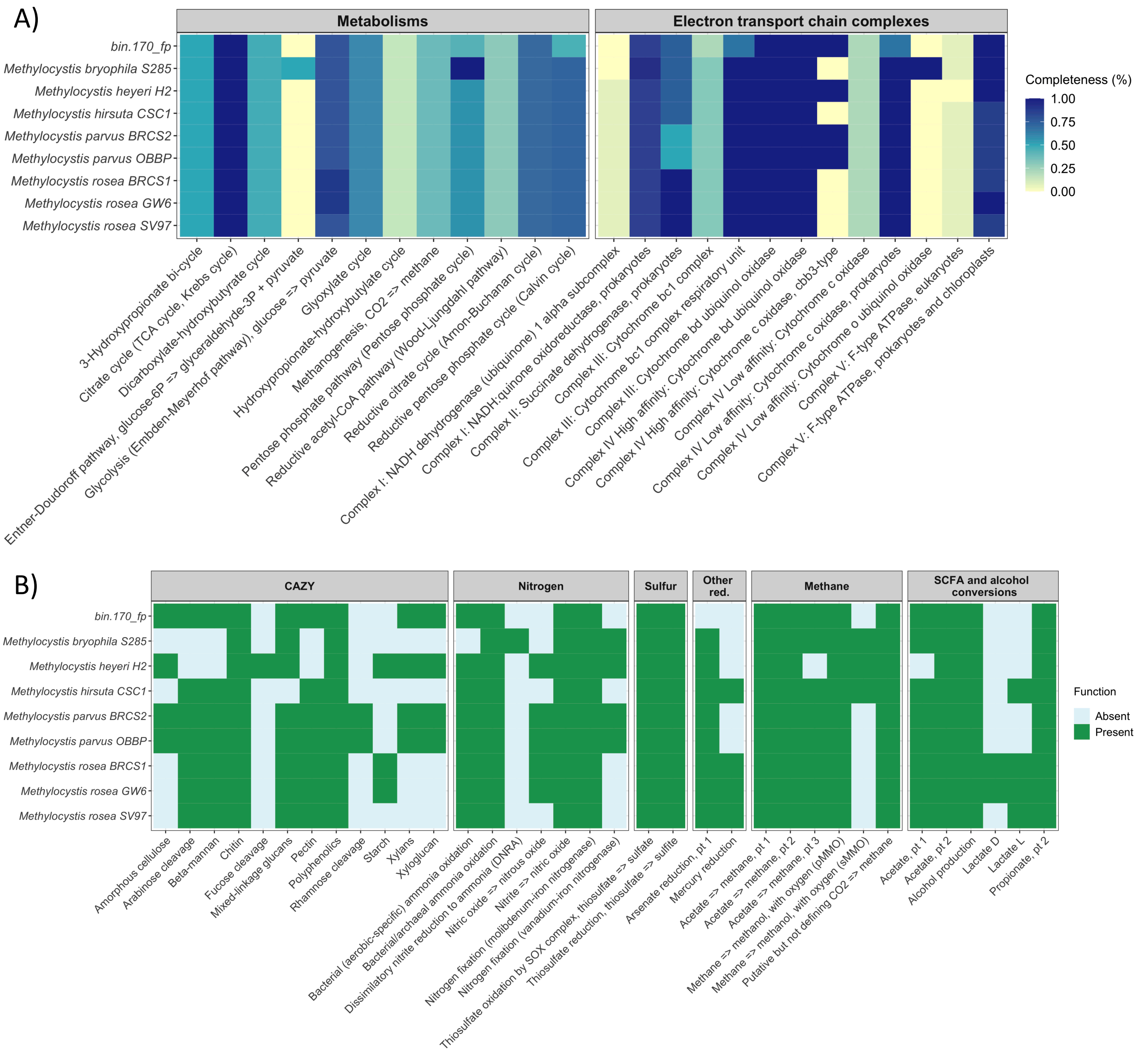

Supplementary Figure S2. DRAM annotations of bin.170_fp and the reference genomes. (A) Metabolism pathways and electron transport chain complexes. The colors in the heatmap represent their completeness in each genome. (B) Presence and absence of metabolic functions. The colors in the heatmap represent their presence or absence in each genome.

2 Supplementary Tables

Supplementary Table S1. Optimized parameters used for each analyzing step on the KBase platform.

Step	Software	Version	Parameters
Filter and Trim	FastQC	0.11.5	Default
	Trimmomatic	0.36	Adapters: TruSeq3-PE-2; Minimum read lenght: 70 bp; Minimum quality: 20
Taxonomic Classification (Reads)	Kaiju	1.7.3	Reference database: NCBI Blast nr (no Euks); Low abundance filter: 0.01%; Subsample percent: 100%;
Contigs Assembly	Merge Reads Libraries	1.0.1	Default
	MEGAHIT	1.2.9	Meta-large assembly; Minimum contig length: 2000 bp
Binning	MetaBat2	1.7	Minimum contig length: 2000 bp
	Maxbin2	2.2.4	Probability threshold: 0.9; Marker set: 107 and 40; Minimum contig length: 2000 bp
Quality Check	CheckM	1.0.18	Full reference tree
Taxonomic Classification (MAGs)	GTDB-Tk	1.1.0	Default
Functional Annotation	DRAM	0.0.2	Minimum contig length: 2000 bp
Phylogenomic Tree	Build Microbial SpeciesTree	1.6.0	Default
Mapping of the bin.170_fp in the metagenome	Bowtie2	2.3.2	Alignment Type: end-to-end; Alignment Type Preset Options: very-sensitive

Supplementary Table S2. Reference genomes used for phylogenomic and pan-genomic analyses.

Reference genome	BioProject	BioSample	NCBI link	Access date
Methylocystis bryophila S285	PRJNA376255	SAMN06368462	https://www.ncbi.nlm.nih.gov/assembly/GCF_002117405.1	10 May 2022
Methylocystis heyeri H2	PRJNA528078	SAMN11168656	https://www.ncbi.nlm.nih.gov/assembly/GCF_004802635.2	10 May 2022
Methylocystis hirsuta CSC1	PRJNA487728	SAMN09908883	https://www.ncbi.nlm.nih.gov/assembly/GCF_003722355.1	10 May 2022
Methylocystis parvus BRCS2	PRJNA565504	SAMN12748973	https://www.ncbi.nlm.nih.gov/assembly/GCF_009685195.1	10 May 2022
Methylocystis parvus OBBP	PRJNA81429	SAMN02469457	https://www.ncbi.nlm.nih.gov/assembly/GCF_000283235.1	10 May 2022
Methylocystis rosea BRCS1	PRJNA565504	SAMN12748972	https://www.ncbi.nlm.nih.gov/assembly/GCF_009685175.1	10 May 2022
Methylocystis rosea GW6	PRJNA506289	SAMN10457790	https://www.ncbi.nlm.nih.gov/assembly/GCF_003855495.1	10 May 2022
Methylocystis rosea SV97	PRJNA165573	SAMN02256431	https://www.ncbi.nlm.nih.gov/assembly/GCF_000372845.1	10 May 2022

Supplementary Table S3. List of the 49 universal genes defined by Clusters of Orthologous Groups (COG) gene families used for the phylogenomic analysis.

COG	Genes	Related-functions
COG0012	COG0012	Predicted GTPase, probable translation factor [Translation, ribosomal structure and biogenesis]
COG0013	AlaS	Alanyl-tRNA synthetase [Translation, ribosomal structure and biogenesis]
COG0016	PheS	Phenylalanyl-tRNA synthetase alpha subunit [Translation, ribosomal structure and biogenesis]
COG0018	ArgS	Arginyl-tRNA synthetase [Translation, ribosomal structure and biogenesis]
COG0030	KsgA	Dimethyladenosine transferase (rRNA methylation) [Translation, ribosomal structure and biogenesis]
COG0041	PurE	Phosphoribosylcarboxyaminoimidazole (NCAIR) mutase [Nucleotide transport and metabolism]
COG0046	PurL	Phosphoribosylformylglycinamidine (FGAM) synthase. synthetase domain [Nucleotide transport and metabolism]
COG0048	RpsL	Ribosomal protein S12 [Translation, ribosomal structure and biogenesis]
COG0049	RpsG	Ribosomal protein S7 [Translation, ribosomal structure and biogenesis]
COG0051	RpsJ	Ribosomal protein S10 [Translation, ribosomal structure and biogenesis]
COG0052	RpsB	Ribosomal protein S2 [Translation, ribosomal structure and biogenesis]
COG0072	PheT	Phenylalanyl-tRNA synthetase beta subunit [Translation, ribosomal structure and biogenesis]
COG0080	RplK	Ribosomal protein L11 [Translation, ribosomal structure and biogenesis]
COG0081	RplA	Ribosomal protein L1 [Translation, ribosomal structure and biogenesis].
COG0082	AroC	Chorismate synthase [Amino acid transport and metabolism].
COG0086	RpoC	DNA-directed RNA polymerase. beta' subunit/160 kD subunit [Transcription]
COG0087	RplC	Ribosomal protein L3 [Translation, ribosomal structure and biogenesis]
COG0088	RplD	Ribosomal protein L4 [Translation, ribosomal structure and biogenesis]
COG0089	RplW	Ribosomal protein L23 [Translation, ribosomal structure and biogenesis]
COG0090	RplB	Ribosomal protein L2 [Translation, ribosomal structure and biogenesis]
COG0091	RplV	Ribosomal protein L22 [Translation, ribosomal structure and biogenesis]
COG0092	RpsC	Ribosomal protein S3 [Translation, ribosomal structure and biogenesis]
COG0093	RplN	Ribosomal protein L14 [Translation, ribosomal structure and biogenesis]
COG0094	RplE	Ribosomal protein L5 [Translation, ribosomal structure and biogenesis]
COG0096	RpsH	Ribosomal protein S8 [Translation, ribosomal structure and biogenesis]
COG0097	RplF	Ribosomal protein L6P/L9E [Translation, ribosomal structure and biogenesis]
COG0098	RpsE	Ribosomal protein S5 [Translation, ribosomal structure and biogenesis]
COG0099	RpsM	Ribosomal protein S13 [Translation, ribosomal structure and biogenesis]
COG0100	RpsK	Ribosomal protein S11 [Translation, ribosomal structure and biogenesis]

Supplementary Table S3 (continuation). List of the 49 universal genes defined by Clusters of Orthologous Groups (COG) gene families used for the phylogenomic analysis.

COG	Genes	Related-functions
COG0102	RplM	Ribosomal protein L13 [Translation, ribosomal structure and biogenesis]
COG0103	RpsI	Ribosomal protein S9 [Translation, ribosomal structure and biogenesis]
COG0105	Ndk	Nucleoside diphosphate kinase [Nucleotide transport and metabolism]
COG0126	Pgk	3-phosphoglycerate kinase [Carbohydrate transport and metabolism]
COG0127	COG0127	Xanthosine triphosphate pyrophosphatase [Nucleotide transport and metabolism]
COG0130	TruB	Pseudouridine synthase [Translation, ribosomal structure and biogenesis]
COG0150	PurM	Phosphoribosylaminoimidazole (AIR) synthetase [Nucleotide transport and metabolism]
COG0151	PurD	Phosphoribosylamine-glycine ligase [Nucleotide transport and metabolism]
COG0164	RnhB	Ribonuclease HII [DNA replication, recombination. and repair]
COG0172	SerS	Seryl-tRNA synthetase [Translation, ribosomal structure and biogenesis]
COG0185	RpsS	Ribosomal protein S19 [Translation, ribosomal structure and biogenesis]
COG0186	RpsQ	Ribosomal protein S17 [Translation, ribosomal structure and biogenesis]
COG0215	CysS	Cysteinyl-tRNA synthetase [Translation, ribosomal structure and biogenesis]
COG0244	RplJ	Ribosomal protein L10 [Translation, ribosomal structure and biogenesis]
COG0256	RplR	Ribosomal protein L18 [Translation, ribosomal structure and biogenesis]
COG0343	Tgt	Queuine/archaeosine tRNA-ribosyltransferase [Translation, ribosomal structure and biogenesis]
COG0504	PyrG	CTP synthase (UTP-ammonia lyase) [Nucleotide transport and metabolism]
COG0519	GuaA	GMP synthase. PP-ATPase domain/subunit [Nucleotide transport and metabolism]
COG0532	InfB	Translation initiation factor 2 (IF-2; GTPase) [Translation, ribosomal structure and biogenesis]
COG0533	QRI7	Metal-dependent proteases with possible chaperone activity [Posttranslational modification, protein turnover, chaperones]

Supplementary Table S4. List of the genes recovered from pan-genomic analysis used for the metabolic model and flagellum assembly predictions.

Metabolism	Gene	Function	Kegg ID
Methane oxidation	pmoA-amoA	methane/ammonia monooxygenase subunit A	K10944
Methane oxidation	pmoB-amoB	methane/ammonia monooxygenase subunit B	K10945
Methane oxidation	pmoC-amoC	methane/ammonia monooxygenase subunit C	K10946
Methane oxidation	xoxF	lanthanide-dependent methanol dehydrogenase	K23995
Formaldehyde assimilation, serine pathway	AGXT	alanine-glyoxylate transaminase / serine-glyoxylate transaminase / serine-pyruvate transaminase	K00830
Formaldehyde assimilation, serine pathway	hprA	glycerate dehydrogenase	K00018
Formaldehyde assimilation, serine pathway	gckA	glycerate 2-kinase	K11529
Formaldehyde assimilation, serine pathway	eno	enolase	K01689
Formaldehyde assimilation, serine pathway	ppc	phosphoenolpyruvate carboxylase	K01595
Formaldehyde assimilation, serine pathway	mdh	malate dehydrogenase	K00024
Formaldehyde assimilation, serine pathway	mtkB	malate-CoA ligase subunit alpha	K08692
Formaldehyde assimilation, serine pathway	mtkA	malate-CoA ligase subunit beta	K14067
Formaldehyde assimilation, serine pathway	mcl	malyl-CoA/(S)-citramalyl-CoA lyase	K08691
Formaldehyde assimilation, serine pathway	glyA	glycine hydroxymethyltransferase	K00600
Formaldehyde assimilation, serine pathway	gpmA	2,3-bisphosphoglycerate-dependent phosphoglycerate mutase	K01834
Formaldehyde assimilation, serine pathway	serA	D-3-phosphoglycerate dehydrogenase / 2-oxoglutarate reductase	K00058
Formaldehyde assimilation, serine pathway	serC	phosphoserine aminotransferase	K00831
Formaldehyde assimilation, serine pathway	serB	phosphoserine phosphatase	K01079
Formaldehyde oxidation	fae	5,6,7,8-tetrahydromethanopterin hydro-lyase	K10713
Formaldehyde oxidation	mtdB	methylene-tetrahydromethanopterin dehydrogenase	K10714
Formaldehyde oxidation	mch	methenyltetrahydromethanopterin cyclohydrolase	K01499
Formaldehyde oxidation	ftr	formylmethanofuran--tetrahydromethanopterin N-formyltransferase	K00672
Formaldehyde oxidation	fwdA	formylmethanofuran dehydrogenase subunit A	K00200
Formaldehyde oxidation	fwdB	formylmethanofuran dehydrogenase subunit B	K00201

Supplementary Table S4 (continuation). List of the genes recovered from pan-genomic analysis used for the metabolic model and flagellum assembly predictions.

Metabolism	Gene	Function	Kegg ID
Formaldehyde oxidation	fwdC	formylmethanofuran dehydrogenase subunit C	K00202
Formaldehyde oxidation	fdoG	ormate dehydrogenase major subunit	K00123
Formaldehyde oxidation	fdoH	formate dehydrogenase iron-sulfur subunit	K00124
Formaldehyde oxidation	fdoI	formate dehydrogenase subunit gamma	K00127
Formaldehyde oxidation	fdsD	formate dehydrogenase subunit delta	K00126
Formaldehyde oxidation	mtdA	methylenetetrahydrofolate/methylenetetrahydromethanopterin dehydrogenase (NADP+)	K00300
Formaldehyde oxidation	fchA	methenyltetrahydrofolate cyclohydrolase	K01500
Formaldehyde oxidation	fhs	formate--tetrahydrofolate ligase	K01938
Ethylmalonyl pathway	acs	acetyl-CoA synthetase	K01895
Ethylmalonyl pathway	atoB	acetyl-CoA C-acetyltransferase	K00626
Ethylmalonyl pathway	phbB	acetoacetyl-CoA reductase	K00023
Ethylmalonyl pathway	croR	3-hydroxybutyryl-CoA dehydratase	K17865
Ethylmalonyl pathway	ccr	crotonyl-CoA carboxylase/reductase	K14446
Ethylmalonyl pathway	epi	methylmalonyl-CoA/ethylmalonyl-CoA epimerase	K05606
Ethylmalonyl pathway	ecm	ethylmalonyl-CoA mutase	K14447
Ethylmalonyl pathway	mcd	(2S)-methylsuccinyl-CoA dehydrogenase	K14448
Ethylmalonyl pathway	mch	2-methylfumaryl-CoA hydratase	K14449
Ethylmalonyl pathway	mcl	malyl-CoA/(S)-citramalyl-CoA lyase	K08691
Ethylmalonyl pathway	pccA	propionyl-CoA carboxylase alpha chain	K01965
Ethylmalonyl pathway	pccB	propionyl-CoA carboxylase beta chain	K01966
Ethylmalonyl pathway	ecm	ethylmalonyl-CoA mutase	K14447
Ethylmalonyl pathway	mtkB	malate-CoA ligase subunit alpha	K08692
Citrate cycle	mdh	malate dehydrogenase	K00024
Citrate cycle	fumAB	fumarate hydratase, class I	K01676

Supplementary Table S4 (continuation). List of the genes recovered from pan-genomic analysis used for the metabolic model and flagellum assembly predictions.

Metabolism	Gene	Function	Kegg ID
Citrate cycle	fumC	fumarate hydratase, class II	K01679
Citrate cycle	frdA	succinate dehydrogenase / fumarate reductase, flavoprotein subunit	K00239
Citrate cycle	frdB	succinate dehydrogenase / fumarate reductase, iron-sulfur subunit	K00240
Citrate cycle	frdC	succinate dehydrogenase / fumarate reductase, cytochrome b subunit	K00241
Citrate cycle	frdD	succinate dehydrogenase / fumarate reductase, membrane anchor subunit	K00242
Citrate cycle	sucD	succinyl-CoA synthetase alpha subunit	K01902
Citrate cycle	sucC	succinyl-CoA synthetase beta subunit	K01903
Citrate cycle	sucB	2-oxoglutarate dehydrogenase E2 component (dihydrolipoamide succinyltransferase)	K00658
Citrate cycle	sucA	2-oxoglutarate dehydrogenase E1 component	K00164
Citrate cycle	pdhD	dihydrolipoamide dehydrogenase	K00382
Citrate cycle	icd	isocitrate dehydrogenase	K00031
Citrate cycle	acnA	aconitate hydratase	K01681
Citrate cycle	gltA	citrate synthase	K01647
Nitrogen fixation	nifD	nitrogenase molybdenum-iron protein alpha chain	K02586
Nitrogen fixation	nifK	nitrogenase molybdenum-iron protein beta chain	K02591
Nitrogen fixation	nifH	nitrogenase iron protein NifH	K02588
Assimilatory nitrate reduction	nasA	assimilatory nitrate reductase catalytic subunit	K00372
Assimilatory nitrate reduction	nirA	ferredoxin-nitrite reductase	K00366
Nitrification	hao	hydroxylamine dehydrogenase	K10535
Dissimilatory nitrate reduction	nirB	nitrite reductase (NADH) large subunit	K00362
Denitrification	nirK	nitrite reductase (NO-forming)	K00368
Assimilatory sulfate reduction	cysN	sulfate adenylyltransferase subunit 1	K00956
Assimilatory sulfate reduction	cysD	sulfate adenylyltransferase subunit 2	K00957
Assimilatory sulfate reduction	cysC	bifunctional enzyme CysN/CysC	K00955

Supplementary Table S4 (continuation). List of the genes recovered from pan-genomic analysis used for the metabolic model and flagellum assembly predictions.

Metabolism	Gene	Function	Kegg ID
Assimilatory sulfate reduction	cysH	phosphoadenosine phosphosulfate reductase	K00390
Assimilatory sulfate reduction	cysI	sulfite reductase (NADPH) hemoprotein beta-component	K00381
Cysteine biosynthesis	cysK	cysteine synthase	K01738
Ammonium transporter	amtB	ammonium transporter	K03320
Nitrate/nitrite transporter	nrtA	nitrate/nitrite transport system substrate-binding protein	K15576
Nitrate/nitrite transporter	nrtB	nitrate/nitrite transport system permease protein	K15577
Nitrate/nitrite transporter	nrtC	nitrate/nitrite transport system ATP-binding protein	K15578
Sulfate/Thiosulfate transporters	cysU	sulfate/thiosulfate transport system permease protein	K02046
Sulfate/Thiosulfate transporters	sbp	sulfate/thiosulfate transport system substrate-binding protein	K23163
Sulfate/Thiosulfate transporters	cysW	sulfate/thiosulfate transport system permease protein	K02047
Sulfate/Thiosulfate transporters	cysA	sulfate/thiosulfate transport system ATP-binding protein	K02045
Phosphate transporters	pstS	phosphate transport system substrate-binding protein	K02040
Phosphate transporters	pstC	phosphate transport system permease protein	K02037
Phosphate transporters	pstA	phosphate transport system permease protein	K02038
Phosphate transporters	pstB	phosphate transport system ATP-binding protein	K02036
Molybdate transportes	modA	molybdate transport system substrate-binding protein	K02020
Molybdate transportes	modB	molybdate transport system permease protein	K02018
Molybdate transportes	modC	molybdate transport system ATP-binding protein	K02017
Osmoprotectant	opuC	osmoprotectant transport system substrate-binding protein	K05845
Osmoprotectant	opuBD	osmoprotectant transport system permease protein	K05846
Osmoprotectant	opuA	osmoprotectant transport system ATP-binding protein	K05847
Bacterial chemotaxis	MCP	methyl-accepting chemotaxis protein	K03406
Bacterial chemotaxis	cheR	chemotaxis protein methyltransferase	K00575
Bacterial chemotaxis	cheB	two-component system, chemotaxis family, protein-glutamate methylesterase/glutaminase	K03412

Supplementary Table S4 (continuation). List of the genes recovered from pan-genomic analysis used for the metabolic model and flagellum assembly predictions.

Metabolism	Gene	Function	Kegg ID
Bacterial chemotaxis	cheA	two-component system, chemotaxis family, sensor kinase CheA	K03407
Bacterial chemotaxis	cheW	purine-binding chemotaxis protein	K03408
Bacterial chemotaxis	cheY	two-component system, chemotaxis family, chemotaxis protein CheY	K03413
Flagellum assembly	motB	chemotaxis protein	K02557
Flagellum assembly	motC	chemotaxis protein	K10564
Flagellum assembly	fliE	chemotaxis protein	K02408
Flagellum assembly	fliF	flagellar M-ring protein	K02409
Flagellum assembly	fliI	flagellum-specific ATP synthase	K02412
Flagellum assembly	fliJ	flagellar protein	K02413
Flagellum assembly	fliL	flagellar protein	K02415
Flagellum assembly	fliP	flagellar biosynthesis protein	K02419
Flagellum assembly	fliQ	flagellar biosynthesis protein	K02420
Flagellum assembly	fliR	flagellar biosynthesis protein	K02421
Flagellum assembly	flhA	flagellar biosynthesis protein	K02400
Flagellum assembly	flgA	flagellar basal body P-ring formation protein	K02386
Flagellum assembly	flgB	flagellar basal-body rod protein	K02387
Flagellum assembly	flgC	flagellar basal-body rod protein	K02388
Flagellum assembly	flgD	flagellar basal-body rod modification protein	K02389
Flagellum assembly	flgE	flagellar hook protein	K02390
Flagellum assembly	flgF	flagellar basal-body rod protein	K02391
Flagellum assembly	flgG	flagellar basal-body rod protein	K02392
Flagellum assembly	flgH	flagellar L-ring protein	K02393
Flagellum assembly	flgI	flagellar P-ring protein	K02394
Flagellum assembly	flgJ	peptidoglycan hydrolase	K02395
Flagellum assembly	flgK	flagellar hook-associated protein 1	K02396

Supplementary Table S4 (continuation). List of the genes recovered from pan-genomic analysis used for the metabolic model and flagellum assembly predictions.

Metabolism	Gene	Function	Kegg ID
Flagellum assembly	flgL	flagellar hook-associated protein 3	K02397
Flagellum assembly	fliC	flagellin	K02406
Bacterial secretion system	secD	preprotein translocase subunit SecD	K03072
Bacterial secretion system	secE	preprotein translocase subunit SecE	K03073
Bacterial secretion system	secG	preprotein translocase subunit SecG	K03075
Bacterial secretion system	secY	preprotein translocase subunit SecY	K03076
Bacterial secretion system	secA	preprotein translocase subunit SecA	K03070
Bacterial secretion system	secB	preprotein translocase subunit SecB	K03071
Bacterial secretion system	yajC	preprotein translocase subunit YajC	K03210
Bacterial secretion system	yidC	YidC/Oxa1 family membrane protein insertase	K03217
Bacterial secretion system	ftsY	fused signal recognition particle receptor	K03110
Bacterial secretion system	ffh	signal recognition particle subunit SRP54	K03106
Bacterial secretion system	tatA	sec-independent protein translocase protein TatA	K03116
Bacterial secretion system	tatB	sec-independent protein translocase protein TatB	K03117
Bacterial secretion system	tatC	sec-independent protein translocase protein TatC	K03118
